# 1365. Self-Reported Impacts of COVID-19 Pandemic on Patients with Bronchiectasis and Nontuberculous Mycobacterium Pulmonary Infections: Healthcare Avoidance and Other Impacts Across Delta and Omicron Waves

**DOI:** 10.1093/ofid/ofad500.1202

**Published:** 2023-11-27

**Authors:** Haley Miller, Jeremy Hawkins, Sarah A R Siegel, Arielle G Hernandez, Emily Henkle, Kevin L Winthrop

**Affiliations:** Oregon Health & Science University, Portland, Oregon; Oregon Health and Science University, Hillsboro, Oregon; Oregon Health & Science University, Portland, Oregon; OHSU-PSU School of Public Health, Portland, Oregon; Oregon Health & Science University, Portland, Oregon; OHSU-PSU School of Public Health, Portland, Oregon

## Abstract

**Background:**

The COVID-19 pandemic greatly impacted the lives of people across the globe. We sought to understand how the pandemic was perceived by and impacted the care of people living with chronic respiratory diseases, such as bronchiectasis and nontuberculous mycobacterial (NTM) infections.

**Methods:**

We conducted a prospective online survey of adults with chronic lung disease collecting data on demographics, health status, and the impact of the COVID-19 pandemic on treatment, healthcare access and utilization. Participants were enrolled between 9/2021 and 3/2023. We recruited from outpatient bronchiectasis treatment centers and online forums. Patients completed a self-screening survey to verify meeting eligibility criteria (over age 18, chronic lung disease). Baseline survey data were analyzed by viral variant wave [Delta (9/2021-12/2021) and Omicron (1/2022-3/2023)] and COVID-19 avoidance was compared by political party.

**Results:**

Of 1,027 screened, 627 (61%) were eligible. Of those, 305 (49%) completed the baseline survey and were included. Participants were median age 67, mostly female (n = 275, 90%), and 263 (89%) lived in the United States. 285 (93%) had bronchiectasis and 260 (85%) had NTM infection. Overall, 178 (58%) reported extreme concern about COVID-19. Of 254 (85%) patients reporting available telehealth, 235 (93%) reported a telehealth visit. In-person healthcare avoidance lessened or was similar comparing Delta to Omicron waves, e.g. avoidance of doctor visits (39% vs 29%, p=0.07), procedures (21% vs 13%, p=0.09), and lab tests (13% vs 13%, p=0.9). Participant level of concern about their lung disease remained high 98% to 95% across Delta and Omicron waves (p=0.08), while the proportion of those infected increased with Omicron (Table 2). When stratified by political party, Democrats were more likely to take social distancing precautions and take vaccine than those identifying as Republican (Table 3).

**Figure:**
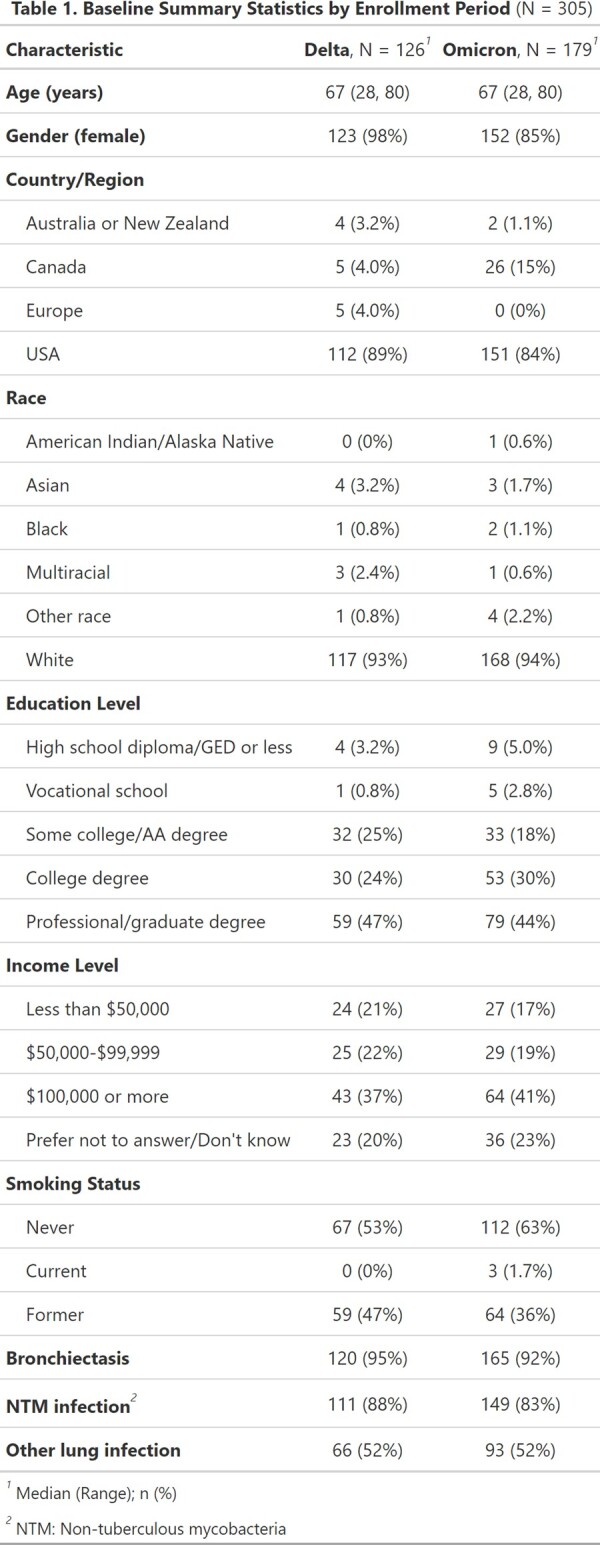

**Figure:**
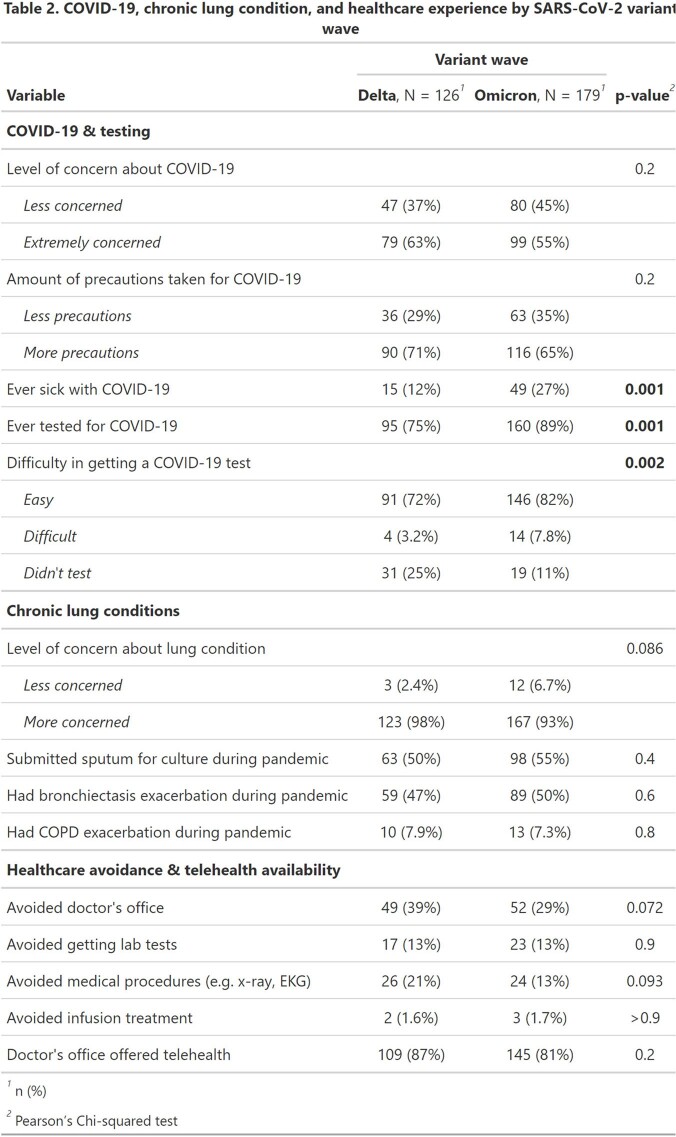

**Figure:**
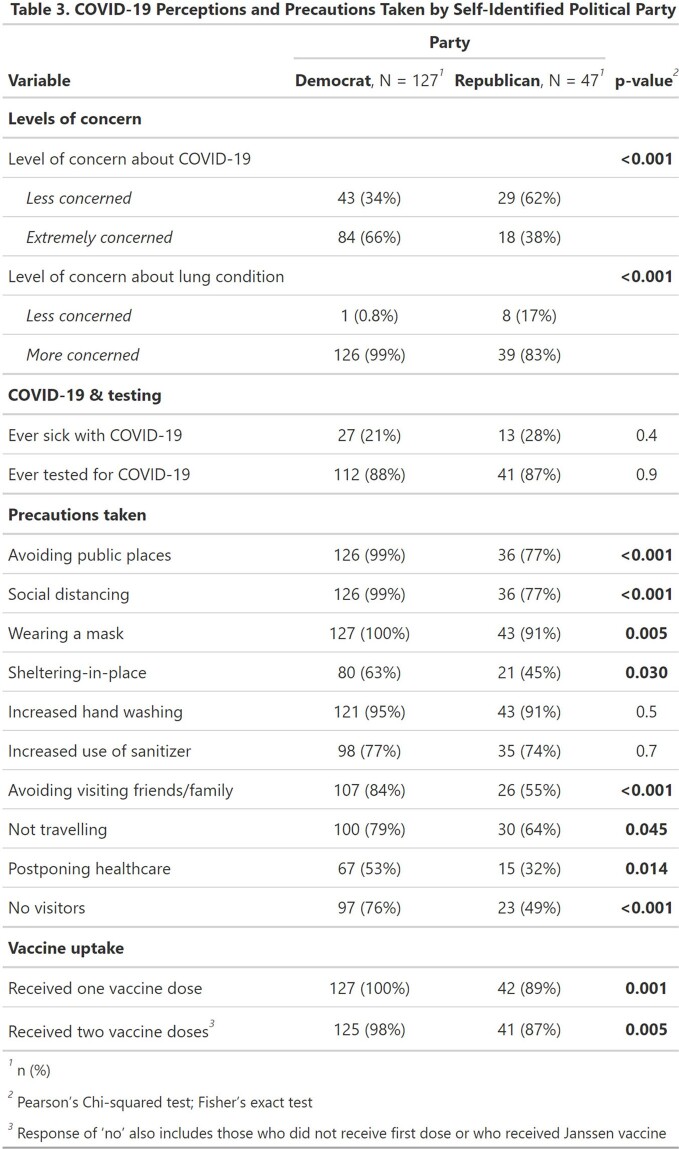

**Conclusion:**

Bronchiectasis and NTM patients reported a high level of concern about COVID-19 and many avoided in-person healthcare during the pandemic, which could impact disease progression. Most individuals took social distancing precautions, but levels varied by political party. This study was funded by Insmed Incorporated in accordance with fair market value.

**Disclosures:**

**Emily Henkle, PhD, MPH**, AN2 Therapeutics: Advisor/Consultant|MannKind: Advisor/Consultant **Kevin L. Winthrop, MD, MPH**, AN2: Advisor/Consultant|AN2: Grant/Research Support|Insmed: Advisor/Consultant|Insmed: Grant/Research Support|Insmed: This study was funded by Insmed Inc.|Paratek: Advisor/Consultant|Paratek: Grant/Research Support|Red Hill Biopharma: Advisor/Consultant|Red Hill Biopharma: Board Member|Red Hill Biopharma: Grant/Research Support|Renovion: Advisor/Consultant|Renovion: Grant/Research Support|Spero: Advisor/Consultant|Spero: Grant/Research Support

